# Oviductal Telocytes in Patients with Uterine Myoma

**DOI:** 10.3390/biomedicines9081060

**Published:** 2021-08-20

**Authors:** Veronika Aleksandrovych, Anna Wrona, Tomasz Bereza, Kazimierz Pityński, Krzysztof Gil

**Affiliations:** 1 Department of Pathophysiology, Jagiellonian University Medical College, 31-121 Krakow, Poland; v.aleksandrovych@uj.edu.pl; 2 Gynecology and Obstetrics Ward with Gynecologic Oncology Subdivision, J.Śniadecki’s Specialistic Hospital, 33-300 Nowy Sącz, Poland; annawrona788@gmail.com; 3 Department of Anatomy, Jagiellonian University Medical College, 31-034 Krakow, Poland; tomasz.1.bereza@uj.edu.pl; 4 Department of Gynecology and Oncology, Jagiellonian University Medical College, 31-501 Krakow, Poland; kazimierz.pitynski@uj.edu.pl

**Keywords:** telocytes, oviduct, infertility, muscular contractility, CD34, PDGFRα

## Abstract

Tubal factor infertility occurs in 30–35% of infertile pairs and may be caused by impaired muscular contractility and ciliary beating as well as immunological imbalance and chronic inflammation. Newly discovered telocytes (TCs) have a wide palette of features, which play a role in oviduct physiology. We have observed tissue samples from human fallopian tubes in patients with and without uterine myoma by immunolabelling. According to the immunohistochemical co-expression of markers, it has been determined that TCs are engaged in a wide range of physiological processes, including local innervation, sensitivity to hypoxia, regulation of calcium, and sex steroid hormones balances. Due to the proximity of NOS- and ChAT-positive nerve fibers and the expression of ion channels markers, tubal TCs might be considered conductor cells. Additionally, their integration in contractions and cilia physiology in the context of fertility has been revealed. We have observed the difference in telocytes expression in the human oviduct between groups of patients and attempted to describe this population of cells specifically in the case of infertility development, a clinically relevant avenue for further studies.

## 1. Introduction

The oviduct (known as the fallopian tube in humans) is an essential part of the female reproductive system in the context of fertility [1], which consists of four anatomical parts (from lateral to medial): The infundibulum and associated fimbriae near the ovary, the ampulla, the isthmus, which is closest to the uterus, and the part penetrating the myometrium (intramural or interstitial portion) [2,3,4]. There are several histologically distinct types of tissue in the oviduct: Serosa, smooth muscle, subserosa, lamina propria, and mucosal layers [2,5]. The inner layer, the mucosa, includes two types of cells: Mucosal and ciliated cells, that have a direct sensory role [2] and are controlled by sex steroid hormones, growth factors (for instance, epidermal growth factor-EGF), and neuronal stimulation [6,7]. Many pathological conditions associated with infertility and ectopic pregnancies have been shown either to destroy cilia or to reduce ciliary motion, or both [1,7,8]. The local immune system of the fallopian tube is presented by different types of cells, reflecting a balance between innate and adaptive immune cells [9,10], which emphasizes their role in the immune response. The main function of the human oviduct is the transportation of ova, sperm, and embryo due to muscular contractions, ciliary beating, and the flow of tubal secretions [1]. All mentioned processes are controlled by sex hormones, nerves, growth factors, and other paracrine signals [11]. Sufficient expression of estrogen receptor alpha in the isthmus of the oviduct and normal progesterone levels allow for the successful transport of the embryo from the Fallopian tube to the uterus [12].

Nowadays, the tubal factor is detected in about 30–35% of infertile women [7]. The main reasons for this kind of infertility are oviduct blockage, congenital malformations, pelvic inflammatory disease (the most common factor), acute salpingitis, endometriosis, former surgical treatment leading to adhesions, polyps, diverticula of the fallopian tubes, hydrosalpinx, and hormonal imbalances [7,13]. Women, affected by uterine myoma (the most widespread benign pathology in the uterus), often have infertility and not always only myoma-related. We want to focus our attention on two important physiological processes that occur within the human fallopian tube and that are crucial for pregnancy: Muscular contractility and ciliary motility. Doubtlessly, both processes are important for ovum transport; however, they may occur separately without any harmful effect on the result. The absence of myometrial contractility does not always lead to the dysfunction of ciliar motility (for instance in rats and rabbits) [14,15]. According to published data, inhibition of nitric oxide synthase (NO) in the oviduct stimulates tubal motility and accelerates ovum transport [16,17].

Over the last ten years, telocytes (TCs) have been discussed by several researchers worldwide. The phenomenon of these cells is the multifaceted nature of its properties on the one hand, and on the other, a matter of debate because of similarity with other cells by the origin of immunohistochemical profile. Typical telocytes are of mesenchymal origin, having a small oval-shape body with extremely long cellular prolongations named telopodes (Tps) with alternating thin segments (podomers) and dilated segments (podoms) [18,19]. These cells have been described in more than fifty anatomical units in human and animal bodies (fish, reptiles, birds, and mammals) [20]. They are different from other mesenchymal cells due to their own morphology, immunohistochemical and secretome profiles, gene expression, and mRNA levels [21,22]. TCs make homo- and heterocellular contacts with plenty of surrounding cells and anatomical units such as blood vessels, nerve fibers, or stem cells, underlining their importance in tissue homeostasis. Transmission electron microscopy (TEM) and immunolabelling is commonly used for the primary identification of TCs. Despite the fact that we do not have a single unique marker for TCs, the combination of CD34 and platelet-derived growth factor receptor alpha (PDGFRα) are the most commonly used markers for telocyte detection among the molecules that exhibit immunopositivity in these cells [19,20,21,22,23]. TCs also have receptors for growth factors and sex steroids. They are involved in the electrical modulation of excitable tissue and play a role in fibrosis and repairing [21,22,23,24].

The Popescu L.M. and Ciontea S.M. scientific groups were the first to describe tubal telocytes, their localization, and the amount in different layers of the fallopian tube in 2005 (Table 1) [7,24,25,26]. 

Oviduct TCs express estrogen/progesterone receptors and thus might act as “hormonal sensors” [7,18,26,27]. According to the literature, c-kit, vimentin, CD34, and PDGFRα were used for the primary identification of tubal TCs [24,25,26,27]. Several studies have reported a role of TCs in immune surveillance or in morphogenetic bioelectrical signaling [26,28,29].

**Figure 1:**
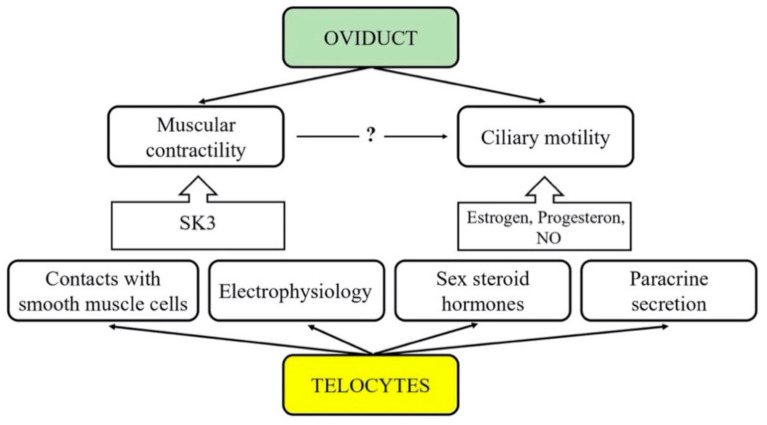
Interactions between telocytes and primary physiological processes within Fallopian tube.

The aim of our study is to provide new insight on the involvement of tubal telocytes in the development of infertility through a direct and indirect impact on muscular contractility and ciliary motility in the human oviduct (Figure 1). Although the exact reason for the observed association between tubal TCs and pathophysiological mechanisms of infertility remains unclear, we intend to reveal possible correlations essential for a more thorough explanation. We also intend to compare the correlation between oviductal telocytes and the presence of uterine myoma.

## 2. Materials and Methods

### 2.1. Subjects

The study group is comprised of eight patients with uterine myoma (mean age 52.9 ± 5.9 years), while the control group by nine patients without uterine myoma (mean age 59.3 ± 9 years). We retrospectively analyzed the data using medical record notes from all patients. The study group was characterized: One patient had 4 labors, one patient had 2 labors, three patients had 1 labor, three patients had zero labors. Miscarriage was noted only in one patient. The control group: One patient had 6 labors; one patient had 5 labors; one patient had 3 labors; three patients had 2 labors; one patient had 2 labors; one patient had 1 labor, and one woman was without any pregnancies. Miscarriage was noted in two patients, who had more than two labors. All women undergoing a laparoscopic hysterectomy were enrolled into the current study. Samples of tissue from the ampullar part of both oviducts (left and right) from all patients with and without uterine myoma were taken for further observation. In total, thirty-four oviducts were observed in the current study. Postsurgical histological examination of the uterus and fallopian tubes did not reveal any signs of malignant tumors.

### 2.2. Ethical Approval

The study was conducted in accordance with the moral, ethical, regulatory, and scientific principles governing clinical research. All surgical samples were retrieved with the approval of the Jagiellonian University Bioethical Committee using procedures that conformed to the guidelines of the Declaration of Helsinki (Protocol No. 1072.6120.48.2018).

### 2.3. Tissue Processing

Fresh hysterectomy specimens were collected and rinsed thoroughly with PBS (phosphate-buffered saline, 0.01 M, pH = 7.4), fixed in 4% phosphate-buffered paraformaldehyde, routinely processed, and embedded in paraffin. Serial sections were cut and mounted on poly-L-lysine-coated glass slides.

### 2.4. Routine Histology

The sections were deparaffinized, rehydrated and stained with either hematoxylin–eosin (H&E) to evaluate the gross tissue organization or Masson trichrome staining to detect collagen deposits.

### 2.5. Immunofluorescence

After deparaffinization and rehydration, the slides were incubated for 30 min in PBS with the appropriate normal serum and 0.3% Triton X-100 (Sigma, St. Louis, MO, USA) at room temperature, followed by overnight incubation at 4°C in a solution of PBS with the appropriate normal serum containing a primary antibody (or a mixture of primary antibodies) and 0.3% Triton X-100. After 5 washes (10 min each) in PBS, the specimens were incubated for 1 h at room temperature with a secondary antibody (or a mixture of secondary antibodies) diluted in PBS with the appropriate normal serum and 0.3% Triton X-100. Finally, the slides were washed in two changes (10 min each) of PBS and cover-slipped with a fluorescence mounting medium (Dako, Denmark). Labeled specimens were analyzed immediately. The following primary and secondary antisera were used (Table 2).

### 2.6. Microscopic Examination

Slides were examined using an MN800FL epifluorescence microscope (OptaTech, Warszawa, Poland) equipped with an Olympus DP74 digital CCD camera. Digital images were collected at 200×, 400×, or 600× magnification. The qualitative analysis of cells and nerve fibers was provided in 10 consecutive high-power fields of vision (400×) using the Multiscan 18.03 (CSS, Warsaw, Poland) computer-based image analysis system. All samples were assessed by two independent specialists (each blinded to each other) without any knowledge of clinical parameters or other prognostic factors to avoid bias.

The presence and distribution of immunoreactivity to the PGP 9.5 pan-neuronal marker was evaluated to assess tubal autonomic innervation. Nerve cells and nerve fibers were evaluated on the basis of their morphology. ChAT- and iNOS- immunoreactivity was evaluated to assess the presence and distribution of different populations and subtypes of autonomic nerves. The use of mast cell tryptase staining enabled c-kit-positive mast cells to be distinguished from c-kit-positive TCs. Furthermore, for primary identification of TCs, we used all widely used and proved immunohistochemical combinations of markers as specified in the literature. TCs were considered as cells that were c-Kit positive and tryptase negative concurrently, with their characteristic morphology and distribution in tissue samples [30,31,32]. Additionally, cells positive for CD34 and PDGFRα with their characteristic morphology and localization were also recognized as TCs. Cells double-positive for c-kit and vimentin were also identified as TCs. CD34-positive interstitial cells lacked CD31 immunoreactivity, thus making them clearly distinguishable from CD34-positive/CD31-positive vascular endothelial cells, and were also identified as TCs.

Vascular density was evaluated by analyzing CD31 and sFlt-1 (VEGFR-1) in all tubal samples. Additionally, immunopositivity for estrogen and progesterone receptors has been compared in all tissue samples. The immunopositivity of tissue samples for small-conductance calcium-activated potassium channels isoform 3 (SK3) and hypoxia-inducible factor (HIF)-1 has also been observed.

## 3. Results

### 3.1. General Structure of the Human Oviduct

**Figure 2:**
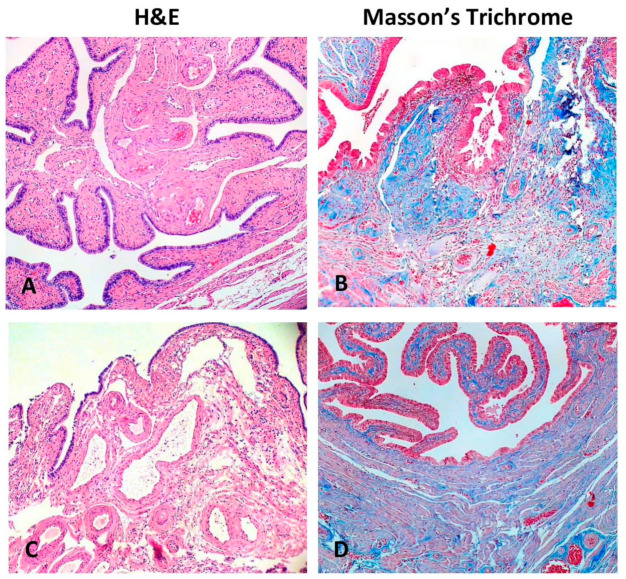
Hematoxylin–eosin and Masson’s trichrome stained sections of the human oviduct in patients with uterine myoma (**C**,**D**) and without (**A**,**B**). Hematoxylin–eosin staining (**A**,**C**) demonstrates the oviduct mucosa. With Masson’s trichrome staining (**B**,**D**), collagen deposits were stained blue and muscle fibers were stained red. The prevalence of collagen deposits is common for the central part of mucosal folds, while in the muscular layer, it was located between muscle bundles. Total magnification: ×100.

Hematoxylin and eosin staining demonstrated that the human oviduct is composed of serosa (adventitia layer), the muscle layer (muscularis mucosa), consisting of an outer longitudinal layer and inner circular layer, and the mucosa. Light microscopy of oviduct tissue using Masson’s trichrome staining was performed for collagen deposit revision and gross evaluation of tissue structure. (Figure 2). Collagen deposits were revealed in the central parts of mucosal folds of the ampulla, while in the muscular layer, they were found to be distributed between muscle bundles. No difference in the gross organization of the oviductal structure in patients with and without uterine fibroid has been revealed.

### 3.2. IHC Analysis of Telocytes in the Fallopian Tubes

**Figure 3:**
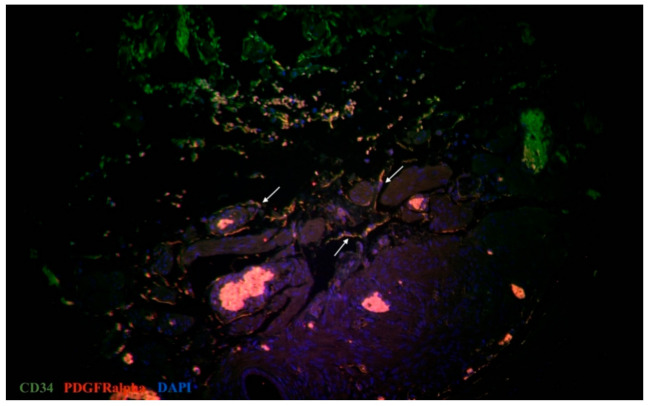
Double immunolabelling of human oviduct stained for PDGFRα (red, Alexa Fluor 594) and CD34 (green, Alexa Fluor 488) in the study group. Nuclei are stained by DAPI. The doubly immunopositive cell with an oval-shaped body and long cellular prolongation was identified as a telocyte (marked by an arrows). Total magnification: ×200.

**Figure 4:**
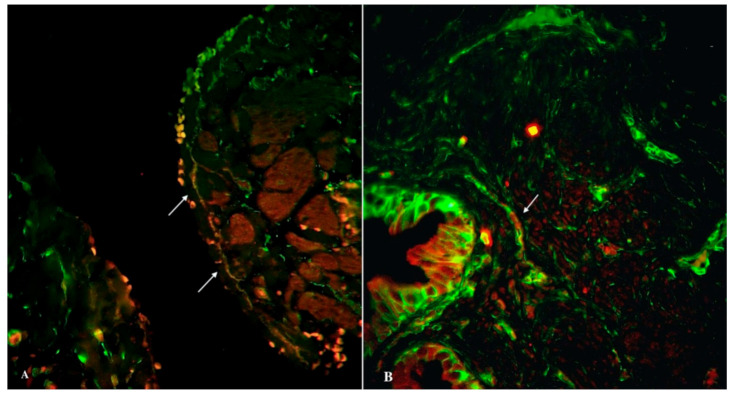
Double immunolabelling of human oviduct stained for c-kit (red, Alexa Fluor 594) and vimentin (green, Alexa Fluor 488) in the control (**A**) and study (**B**) groups. The doubly immunopositive cell with an oval-shaped body and long cellular prolongation was identified as a telocyte (marked by an arrows). Total magnification: ×400.

**Figure 5:**
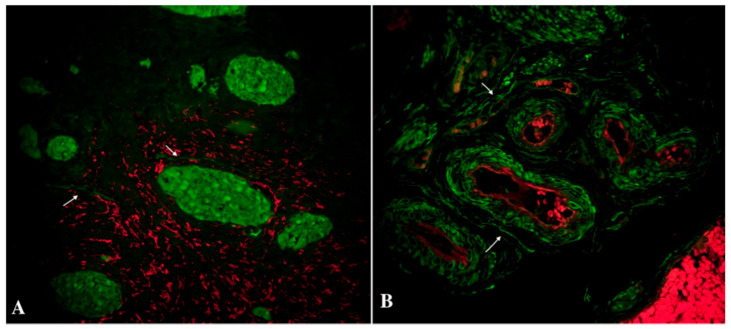
Samples of human oviduct stained for CD31 (red, Alexa Fluor 594) and CD 34 (green, Alexa Fluor 488) in the control (**A**) and study (**B**) groups. The doubly immunopositive structure was identified as vessel, while the cell with an elongated body located between muscle fibers and close to the blood vessel was identified as a telocyte (one of them is indicated by arrows on the image). The telocytes on the right image are located close to blood vessels of different calibers. Double immunostaining has emphasized the difference between cells and anatomical structures (blood vessels). Total magnification: ×400.

Immunofluorescent labeling was performed for the primary identification of TCs in the oviduct. We used all current proven markers, including CD34, PDGFRα, vimentin, and the canonic c-kit. Double immunolabeling for c-kit and tryptase was performed for the identification of mast cells and signs of consequent inflammation. In immunostained slides, c-kit and tryptase double-positive mast cells were generally big and round shaped, with a centrally located nucleus. The c-kit-positive/mast cell tryptase-negative cells were TCs. They have a small body and two-three long cellular prolongations. We also identified telocytes beyond a lamina propria and within the muscular layer (in the vicinity of blood vessels). The same distribution was common for double immunopositive cells for CD34 and PDGFRα (tubal TCs) (Figure 3) and also double immunopositive cells for c-kit and vimentin (tubal TCs) (Figure 4). Immunolabeling for CD31 was performed for the assessment of the vascular density in the tubal tissue. In addition, double immunostaining for CD34/CD31 revealed tubal TCs. Structures with double immunopositivity for CD34 and CD31 were considered to be blood vessels, while those immunopositive for CD34 and immunonegative for CD31 with consequent morphology (sometimes near blood vessels) were considered to be tubal telocytes (Figure 5). In oviductal tissue samples from patients with uterine myoma, we observed more telocytes in comparison with the control group. Oviductal telocytes have the same morphology and localization, but their expression was different in both groups.

### 3.3. IHC Analysis of Nerve Fibers and Its Interactions with Oviduct Telocytes

**Figure 6:**
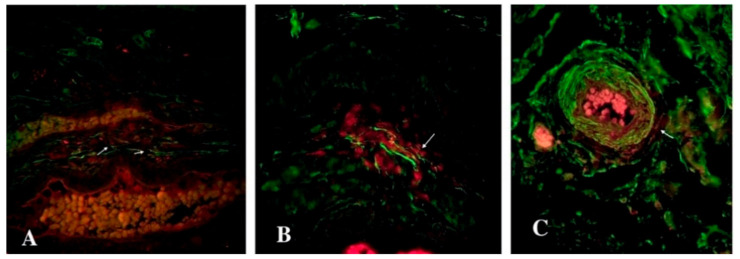
Double immunolabeling of the human oviduct tissue for CD34 (green, Alexa Fluor 488) and PGP 9.5 (red, Alexa Fluor 594)—(**A**); for CD34 and iNOS (red, Alexa Fluor 594)—(**B**); for CD34 and ChAT (red, Alexa Fluor 594)—(**C**). Nerves are presented as red filaments accompanied by green structures (telocytes and blood vessels) longitudinally. Total magnification: ×400.

In the human oviduct PGP 9.5, positive nerve fibers were identified mostly in the interstitial space between smooth muscle cells. They also form a dot-line-like pattern near blood vessels. Some of them interact with CD34-immunopositive tubal telocytes. Thus, we can hypothesize that PGP 9.5—immunopositive structures—interact with smooth muscle cells and telocytes, with a subsequent influence on the muscular motility of the human oviduct. ChAT-immunoreactive nerve fibers were found in the oviduct crypts, beyond the lamina propria, and rarely in the interstitial space of the oviduct. They have been mostly detected close to blood vessels. In crypts, they form networks, sometimes longitudinally. Nerves immunoreactive for iNOS were found throughout all layers of the oviduct samples. The density was higher in the muscular layer and under a lamina propria. Some of the iNOS-immunopositive structures were distributed around large blood vessels. Additional immunostaining for neuronal markers such as iNOS, ChAT, and PGP 9.5 combined with a telocyte marker CD34 revealed doubly immunolabeled focuses, reflecting interactions between nerves and telocytes within the oviduct (Figure 6). The same type of interplay has been described previously in the capsule of uterine myoma within myometrium [33].

### 3.4. IHC Analysis of Estrogen and Progesterone Receptor Expression

**Figure 7:**
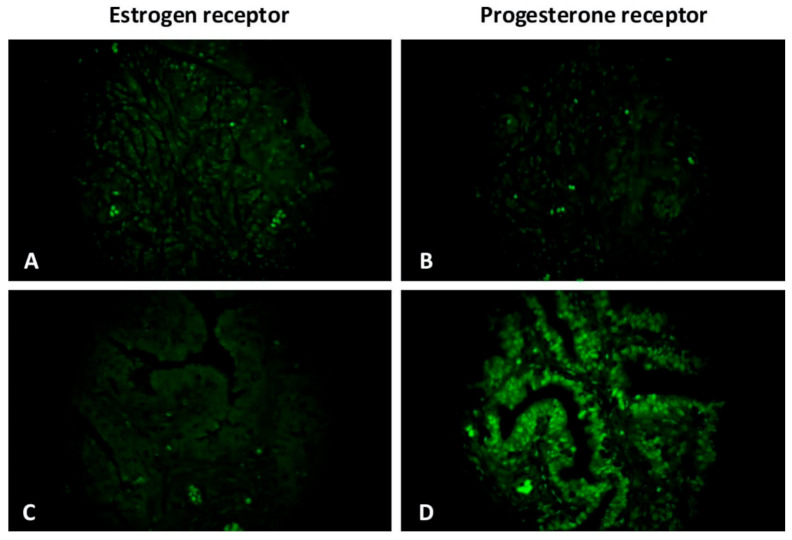
The oviduct tissue (ampulla section) from patients with uterine myoma (**C**,**D**) and without (**A**,**B**) stained for estrogen (**A**,**C**) and progesterone (**B**,**D**) receptors (green, Alexa Fluor 488). Nuclear receptors for both steroid hormones were expressed in the mucosal folds of the oviduct. Total magnification: ×400.

**Figure 8:**
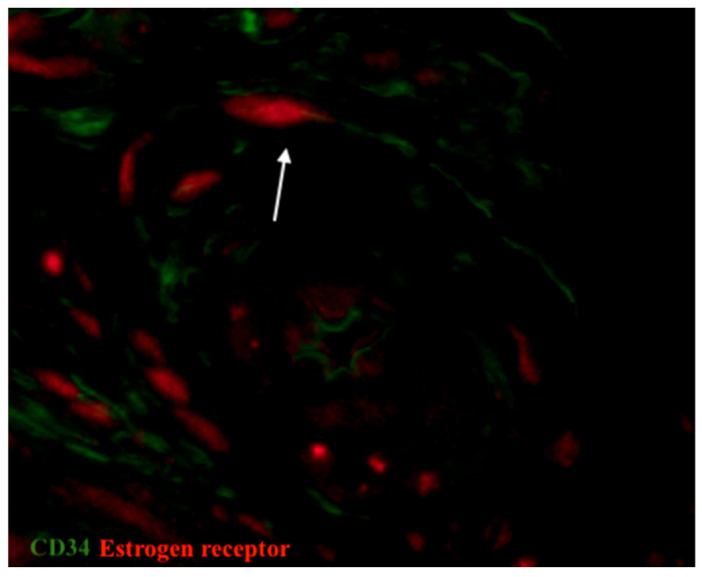
Sample of human oviduct stained for estrogen receptor (red, Alexa Fluor 594) and CD 34 (green, Alexa Fluor 488). A doubly immunopositive telocyte (with red nucleus and green cellular body and prolongations) was identified in the vicinity of a blood vessel in the muscular layer (shown by arrow). Total magnification: ×600.

**Figure 9:**
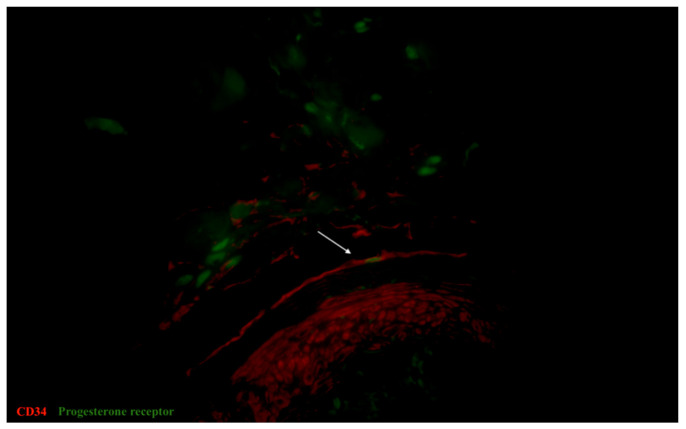
Sample of human oviduct stained for CD34 (red, Alexa Fluor 594) and progesterone receptor (green, Alexa Fluor 488). The cell with immunopositive for CD34 cellular body and two long cellular prolongations, which has an immunopositive nucelus for progesterone, was identified as tubal telocyte (shown by arrow). This double immunopositive cell was revealed in the muscular layer. Total magnification: ×600.

The expression of estrogen and progesterone receptors was detected by IHC. We also performed double immunolabelling for CD34/estrogen receptors as well as CD34/progesterone receptors in the oviduct in order to analyze the role of telocytes, which were doubly positive for both mentioned markers. Generally, in oviductal tissue in both groups, estrogen receptors were mainly observed in the mucosa and more sparingly in the tunica muscularis. In the control group, progesterone receptors were more rarely expressed in comparison with estrogen receptors, but they both have the same distribution (Figure 7). The opposite tendency has been observed in patients with uterine myoma. The expression of progesterone receptors was higher in the study group in comparison with the control, while the expression of estrogen declined. It correlates with the prevalence of progesterone receptors in uterine myoma within the focus of fibrosis and adjacent tissue [34]. In our study, cells doubly positive for CD34/estrogen receptors and CD34/progesterone receptors (tubal telocytes) have been observed mostly in the muscular layer. They were observed close to blood vessels and have elongated oval-shaped bodies (immunopositive for CD34) and nucleus (immunopositive for estrogen and progesterone receptors, respectively) (Figure 8 and Figure 9).

### 3.5. Immunolabelling of Telocytes with Makers of Hypoxia and Ion Channels

**Figure 10:**
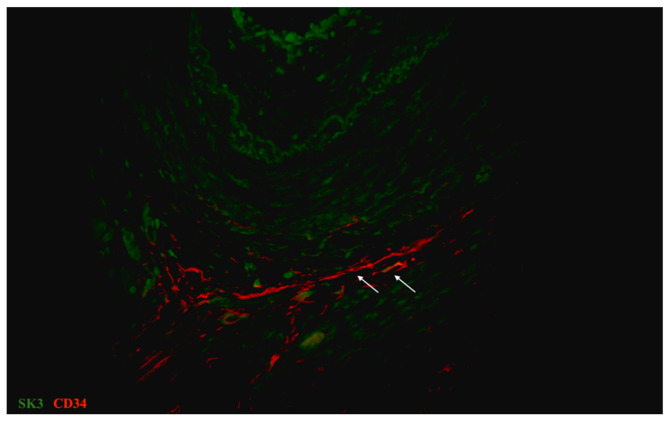
Sample of human oviduct stained for CD34 (red, Alexa Fluor 594) and small-conductance calcium-activated potassium channels isoform 3 (SK3) (green, Alexa Fluor 488). The cell immunopositive for CD34 and SK3 was identified as tubal telocyte (has shown by arrows). This doubly immunopositive cell was observed in the muscular layer. Total magnification: ×400.

Oviductal tissue was characterized by a high expression of small-conductance calcium-activated potassium (SK3) channels throughout the wall with maximal expression in the tunica muscularis. The expression of SK3 was identical in both groups of patients. Double immunolabelling with CD34 and SK3 revealed cells that were positive for both markers and have very long prolongations (Figure 10). Oviductal telocytes were detected between smooth muscle bundles and it seems they may have an effect on muscular contractions in the human oviduct.

**Figure 11:**
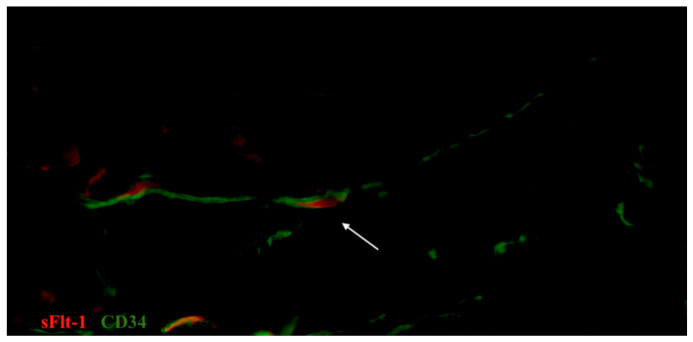
Sample of oviduct stained for sFlt-1 (VEGFR-1) (red, Alexa Fluor 594) and CD34 (green, Alexa Fluor 488). The interaction between telocytes and the expression of the hypoxia marker is shown by the arrow. The high sensitivity of telocytes to hypoxia and their distribution in close vicinity to blood vessels is proved by their co-expression with markers of hypoxia in tissue sample. Total magnification: ×600.

**Figure 12:**
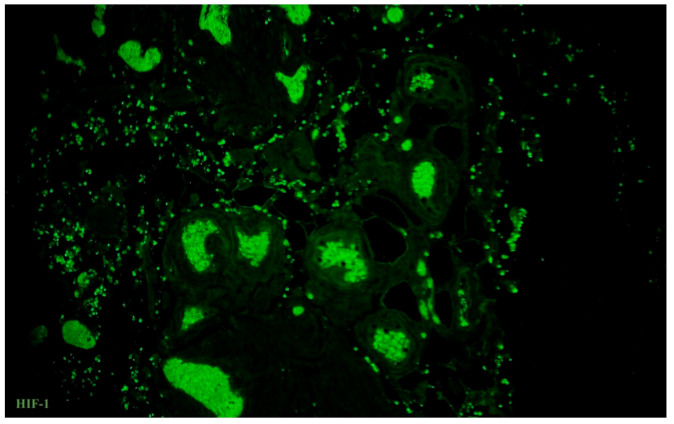
Tissue samples from the ampulla of the human oviduct stained for hypoxia-inducible factor (HIF)-1 (green, Alexa Fluor 488). High expression of HIF-1 was common for all layers of the oviduct in tissue samples from the control group, slightly dominated in outer layers in comparison to the inner. Total magnification: ×200.

Vascular density in the oviduct was evaluated by immunostaining for CD31, while hypoxia was indirectly observed by expression of VEGF receptor-1 (sFlt-1) and hypoxia-inducible factor (HIF)-1. A positive correlation between the density of blood vessels and HIF-1 was observed, while the expression of the anti-angiogenic-related factor (sFlt-1) was lower. Furthermore, double immunolabeling for CD34 and sFlt-1 showed that tubal telocytes have some interaction with the expression of the anti-angiogenic marker due to their vicinity in tissue samples (Figure 11). Of note, HIF-1 was highly expressed in the tunica muscularis and lower in the mucosa, but its expression was common to all layers of the human oviduct in the analyzed tissue samples (Figure 12). The expression of HIF-1 was higher in the oviducts from the control group in comparison with patients with uterine myoma.

## 4. Discussion

Recently, Cretoiu et al. first compared the distribution of TCs in the human fallopian tube. Although there is a definite difference among all histological layers and their respective densities, more cells have been revealed at the border epithelium/lamina propria. The density declined from the inner to the serosa part [24]. A similar tendency has been described by Abd-Abd-Elhafeez et al. in the bovine uterine tube. He divided telocytes into three subpopulations based on size (small, large, and giant telocytes), and stressed that large forms were common also for the lamina propria and epithelium, while giant forms were prevalent in the external layer of the outer perimuscular sheath [35]. We have also observed that more TCs were located in the lamina propria region and less in the tunica muscularis. However, in the muscular layer, they interact with nerves and smooth muscle cells, usually close to blood vessels and co-expressed with markers of hypoxia. It was unexpected for us that oviductal telocytes increases in the co-existence of uterine myoma. In our opinion, it might be explained by their interplay with stem cells and involving the prevention of tissue damage or even regenerative process, which have been discussed by Ibba-Manneschi et al. [36].

Tubal telocytes are positive for sex steroid hormones (estrogen and progesterone) in human and non-human organisms, which are incredibly important for both reproductive systems [27,37,38,39,40]. Yang et al. suggested that they could be involved in dysfunctional tubal motility diseases through decreasing the number and sensitivity of sex steroid receptors after a methotrexate insult in rabbit oviducts [22]. Moreover, estradiol as well as estrogen receptors are indirectly involved in forms of fibrosis such as cardiac, systemic, and renal [41,42]. Through signaling pathways, cell proliferation promotes fibrosis development. Furthermore, these hormones play a role in the extracellular matrix production and may promote B-lymphocytes [37]. The ciliary beating frequency in the fallopian tube is dependent on estrogen and progesterone fluctuations during a menstrual cycle [38]. Low levels of estrogen increase the ciliary beating frequency, while high levels of progesterone weaken their motility. A derivate of progesterone reduces the cilia beating frequency in the fallopian tube without damaging the ciliary morphology [43]. Tubal TCs are positive for both kinds of sex steroid hormones. Hence, they could be like conductor cells, which reflect the fluctuation of hormones during the menstrual cycle and impact motility. To our knowledge, the density of telocytes in myometrium declines during pregnancy [44]. This might be connected with hyperplasia and hypertrophy of myometrial cells but could also be an essential step for preventing myometrial contractions. We hypothesized that a difference in the expression of estrogen and progesterone receptors in layers of the oviduct may also correlate with the density of TCs. In patients with uterine myoma, the density of tubal telocytes is also correlated with a prevalence of progesterone receptors expression. It might stimulate growth factors with mitogenic activity, such as transforming growth factor-β3, basic fibroblast growth factor, epidermal growth factor, and insulin-like growth factor-I [34]. From another point of view, it can lead to local activation of humoral immunity [37] and fibrosis development [41]. This tendency has been observed in our patients of the postmenopausal age group, while the whole mechanism of interaction between the density of tubal telocytes and hormonal misbalance might be conducive to developing tubal factor of infertility.

Tubal motility is also controlled by nerve stimulation. It is crucial for the physiology of this organ and might be destroyed during inflammatory or chronic processes, with clinical effects on fertility. For instance, in women with hydrosalpinx, the density of PGP 9.5-positive nerves was significantly decreased in comparison with women without this condition [11]. Fallopian tubes are sparsely innervated with cholinergic nerve fibers that secrete acetylcholine as a mediator and produce tonic contraction of the oviduct through contacts with smooth muscle cells [45]. Oviductal epithelial cells can produce acetylcholine in a cycle-dependent manner. The choline acetylase (ChAT) immunoreactivity of these cells has been observed in pigs in different stages of the cycle, and it was observed that during the dioestrus and proestrus stage, it is decreased. At prooestrus, ChAT immunoreactivity was confined to ciliated cells. It has been increased during pregnancy. A difference in expression of ChAT-positive neurons has been detected in different parts of the oviduct with high prevalence in infundibulum and decreasing prevalence in the ampulla and isthmus, respectively [46]. *Noreikat* et al. hypothesized that in mice, high oviductal autonomous ciliary activity is independent of the intrinsic cholinergic system and serves to maintain an optimal clearance of the tube throughout all stages of the estrous cycle and early pregnancy [47]. From another point of view, in the murine oviduct, cholinergic receptors play a role in the regulation of intracellular calcium concentration [48]. We observed that tubal TCs interact with PGP 9.5-positive and ChAT-positive nerve fibers in the muscular layer of the oviduct. Indeed, they may regulate intracellular calcium by a different mechanism. Small-conductance calcium-activated potassium (SK3) channels play a role in myometrial contractions. Rosenbaum et al. demonstrated that the expression of SK3 in non-pregnant human myometrium was higher in comparison with that of a pregnant woman. Moreover, SK3 was expressed in TCs in the uterus [49]. In the oviduct, SK3 channels are essential for chloride secretion and, consequently, for fluid formation [50]. Ca^2+^ oscillations, modulated through progesterone and its agonist, stimulate ciliary beating in the mouse oviduct [51]. Tubal TCs express SK3 ion channels and are immunopositive for progesterone receptors, therefore they might have an impact on muscular contractility as well as ciliary beating in the fallopian tube. Ciliary dyskinesia is considered as a risk factor of male and female infertility but is only partly dependent on calcium balance dysregulation [52].

Moreover, NO participates in signal transduction associated with ciliary beating in the oviduct [17]. Nitric oxide synthase is divided into three forms: Neuronal, epidermal, and inducible (iNOS). All of them are expressed in the mouse and human oviduct, while only iNOS can be induced after activation of macrophages or by cytokines (interleukin (IL)-1, IL-2, and IL-12; tumor necrosis factor alpha; and the endotoxin lipopolysaccharide) [16]. iNOS expression leads to the production of NO, a potent dilatator of smooth muscle, by a calcium-independent mechanism. Balance in the iNOS/NO is always important for local homeostasis. In animal models, TCs activated peritoneal macrophages and stimulated production of iNOS [53]. TCs interact with macrophages via the mitochondrial signaling pathway and play a role in local immunosurveillance, especially in endometriosis [54]. This decrease in the density of TCs (in some cases to undetectable levels) correlates with elevated levels of iNOS and other inflammatory markers in rat oviducts affected by endometriosis [55]. We revealed via double immunostaining that tubal TCs interact in some way with NOS-positive nerve fibers. They are co-expressed within the human oviduct. This result was already demonstrated in the human myometrium (affected and unaffected by uterine fibroid) in our previous study [33]. Thus, TCs in the human oviduct might be involved in tubal local homeostasis.

We also want to briefly discuss a possible correlation of tubal TCs and local hypoxia. TCs can stimulate stem cells via paracrine signals [56]. For instance, mouse cardiac TCs secrete interleukin (IL)-6, VEGF, macrophage inflammatory protein 1α (MIP-1α), MIP-2, and monocyte chemoattractant protein-1 (MCP-1), while rat TCs secrete more cytokines: IL-2, IL-10, IL-13, and some chemokines (stimulated by IL-6 signalling) [57]. IL-13 stimulated secretion of VEGF and its receptor (sFlt-1) by oviductal epithelial cells in vivo [58]. In addition, these angiogenic factors could be secreted by oviductal fibroblasts upon stimulation by IL-1 [59]. To our knowledge, TCs are sensitive for angiogenic factors (PDGF and VEGF) and ischemia; they have declined and even disappeared during fibrosis and observed in close vicinity to blood vessels. Our investigation has revealed that tubal TCs and the expression of sFlt-1 do indeed interact. Hypoxia-inducible factor-1α (HIF-1α) is known as a transactivator for the VEGF gene promoter [60] that is also induced by local hypoxia. HIF-1-VEGF signaling is crucial for folliculogenesis in the ovaries, which is also connected with estrogen/progesterone balance. We know that the fluctuation of sex steroid hormones levels is dependent on the menstrual cycle and affects ciliary beating in the oviduct. In addition, it could be responsible for the initiation of (epi)genetic changes in uterine myoma [61]. Abnormal signaling and epigenetic alterations within uterine myoma as well as in the human oviduct provide additional data for clarifying all steps of pathogenesis [62]. For instance, the production of oviductin, the oviduct-specific glycoprotein, differs between the ampulla and the isthmus, has been revealed in the oviduct and the uterus. Its gene expression is regulated by sex steroid hormones [63]. Telocytes have their own genetic and microRNA profiles that reflect their sensitivity to hypoxia. They express a significant amount of pro-angiogenic microRNAs (miR126, miR130a, let-7-family, miR-10, miR-155, miR-503, miR-126, miR-27b, miR-503, and miR-100), as well as miR-21, miR-22, miR-29, and miR-199a, both stromal specific and vascular smooth muscle specific (miR-143/145) [20].

Our study confirmed that tubal TCs are indirectly involved in local angiogenesis as well as particular steps of the pathway, regulated by angiogenic factors, hormones, and paracrine secretion. They are positive for estrogen and progesterone, may stimulate the production of VEGF and its receptor by secreted interleukins, are positive for growth factors receptors (PDGF and VEGF), and interact with the expression of HIF-1 in the human oviduct where it is mostly distributed close to blood vessels in the muscular layer and also near a lamina propria. We also stressed that this population of cells reacts with local inflammation. In oviducts from patients with uterine myoma, which have no gross histological changes, the pool of telocytes has increased. It reflects that telocytes might be considered as the first line of cells, involved in a compensatory way in local homeostatic imbalance. No doubt, further observation is required for a deeper understanding and explanation.

The number of papers related to the tubal infertility factor is relatively limited, mostly due to bioethical issues, including special attention to experiments on pregnant women and organs of the reproductive system and material of human oviducts obtained from patients in the reproductive period.

We are aware of limitations of this research, which has a predominantly observational character, as we assessed the histological samples obtained after a hysterectomy, and the possibility to provide a deep analysis of patient’s medical records in the context of infertility/fertility status was limited. Thus, the conclusions regarding the direct link between oviductal telocytes’ density and infertility are only partially supported by the presented data. We admit that the best way to answer these questions concerning fertility and telocytes relationship would be a long, clinical prospective study. However, even in these two small groups, the difference in “fertility” status was visible. Despite the mean age, retrospective analyses showed that patients from the control group (without uterine myoma) had more pregnancies and labors in comparison with the study group. In some cases, it might be explained by the uterine factor of infertility; however, considering the predominance of other factors of female infertility (tubal and ovarian, not in all.

## 5. Conclusions

The results of this study demonstrate that human oviductal telocytes play their own role in the oviductal microenvironment as well as the pathophysiology of processes, the disturbance of which may lead to infertility (the muscular contractions, innervation, angiogenesis). Oviductal telocytes are positive for estrogen and progesterone receptors, express SK3, and interact with PGP 9.5, iNOS, and ChAT-positive nerve fibers. They are involved in local angiogenesis and secrete VEGF. Of note, the human oviduct in patients with uterine myoma has more telocytes, higher expression of progesterone receptors, and lower expression of HIF-1 in comparison with the control group. Despite the necessity of further research, we are convinced that oviductal telocytes are important for muscular contractions and ciliary motility.

## Figures and Tables

**Table 1:** Distribution of telocytes in the fallopian tube stratums.

Area of the Human Fallopian Tube	Density of TCs (%)
The border epithelium/lamina propria, a ‘belt’ 10 µm thick underneath the basement membrane of the endosalpinx epithelium	18 ± 2
The subepithelial portion of lamina propria (~20 µm thick)	11.7 ± 0.9
Area, containing the whole lamina propria thickness	9
Tunica muscularis	7.8 ± 1.2
Remaining zone beneath serosa	not assessed

**Table 2:** Type, sources, and dilution of antibodies.

Antibody	Catalog Number and Company	Dilution
Primary Antibodies		
Polyclonal rabbit anti-PGP 9.5	Z5116, Dako, Glostrup, Denmark	1:100
Polyclonal mouse anti-NOS	sc-7271, Santa Cruz, Dallas, Texas, USA	1:100
Monoclonal mouse anti-ChAT	sc-55557, Santa Cruz, Dallas, Texas, USA	1:100
Monoclonal mouse anti-CD31	JC70A, Dako, Glostrup, Denmark	1:100
Polyclonal rabbit anti-c-kit	A4502, Dako, Glostrup, Denmark	1:100
Monoclonal mouse anti-CD34	M7165, Dako, Glostrup, Denmark	1:100
Polyclonal goat anti-PDGFR alpha	AF-307-NA, R&D Systems, Minneapolis, Minnesota, USA	1:100
Monoclonal mouse anti-tryptase	M7052, Dako, Glostrup, Denmark	1:100
Monoclonal mouse anti-vimentin	Clone V9, Dako, Glostrup, Denmark	1:50
Monoclonal mouse progesterone-receptor	Clone PgR636, Dako, Glostrup, Denmark	1:100
Monoclonal mouse estrogen receptor	NCL-L-ER-6F11, Leica Biosystems, Newcastle upon Tyne, UK	1:50
Polyclonal goat anti-VEGF R1/Flt-1	AF321, R&D Systems, Minneapolis, Minnesota, USA	5 μg/mL
Monoclonal mouse anti-HIF-1	ab16066, Abcam, Cambridge, UK	1:100
Monoclonal rabbit anti-CD34	ab81289, Abcam, Cambridge, UK	1:200
Polyclonal rabbit anti-KCNN3 (SK3)	APC-025, Alomone Labs, Jerusalem, Israel	1:800
**Secondary Antibodies**		
Alexa Fluor 594 Goat Anti-Mouse	115-585-146, Jackson ImmunoResearch, Ely, UK	1:400
Alexa Fluor 488 Goat Anti-Mouse	115-545-146, Jackson ImmunoResearch, Ely, UK	1:400
Alexa Fluor 594 Goat Anti-Rabbit	111-585-144, Jackson ImmunoResearch, Ely, UK	1:400
Alexa Fluor 488 Rabbit Anti-Mouse	315-545-045, Jackson ImmunoResearch, Ely, UK	1:400
Alexa Fluor 488 Goat Anti-Rabbit	111-545-144, Jackson ImmunoResearch, Ely, UK	1:400
Alexa Fluor 594 Donkey Anti-Goat	705-585-003, Jackson ImmunoResearch, Ely, UK	1:400

## Data Availability

Data is available from authors upon reasonable request.
